# Laying the foundations for a theory of consciousness: the significance of critical brain dynamics for the formation of conscious states

**DOI:** 10.3389/fnhum.2024.1379191

**Published:** 2024-04-26

**Authors:** Joachim Keppler

**Affiliations:** Department of Consciousness Research, DIWISS, Roth, Germany

**Keywords:** conscious processes, universal mechanism, brain dynamics, criticality, phase transition, coherence, zero-point field (ZPF), resonant brain-ZPF coupling

## Abstract

Empirical evidence indicates that conscious states, distinguished by the presence of phenomenal qualities, are closely linked to synchronized neural activity patterns whose dynamical characteristics can be attributed to self-organized criticality and phase transitions. These findings imply that insight into the mechanism by which the brain controls phase transitions will provide a deeper understanding of the fundamental mechanism by which the brain manages to transcend the threshold of consciousness. This article aims to show that the initiation of phase transitions and the formation of synchronized activity patterns is due to the coupling of the brain to the zero-point field (ZPF), which plays a central role in quantum electrodynamics (QED). The ZPF stands for the presence of ubiquitous vacuum fluctuations of the electromagnetic field, represented by a spectrum of normal modes. With reference to QED-based model calculations, the details of the coupling mechanism are revealed, suggesting that critical brain dynamics is governed by the resonant interaction of the ZPF with the most abundant neurotransmitter glutamate. The pyramidal neurons in the cortical microcolumns turn out to be ideally suited to control this interaction. A direct consequence of resonant glutamate-ZPF coupling is the amplification of specific ZPF modes, which leads us to conclude that the ZPF is the key to the understanding of consciousness and that the distinctive feature of neurophysiological processes associated with conscious experience consists in modulating the ZPF. Postulating that the ZPF is an inherently sentient field and assuming that the spectrum of phenomenal qualities is represented by the normal modes of the ZPF, the significance of resonant glutamate-ZPF interaction for the formation of conscious states becomes apparent in that the amplification of specific ZPF modes is inextricably linked with the excitation of specific phenomenal qualities. This theory of consciousness, according to which phenomenal states arise through resonant amplification of zero-point modes, is given the acronym TRAZE. An experimental setup is specified that can be used to test a corollary of the theory, namely, the prediction that normally occurring conscious perceptions are absent under experimental conditions in which resonant glutamate-ZPF coupling is disrupted.

## 1 Introduction

One of the major challenges for cognitive neuroscience lies in deciphering the mechanism behind advanced cognitive processes that culminate in conscious states, with the defining characteristic of consciousness being the presence of phenomenal qualities (qualia). Conscious processes include, on the one hand, stimulus-induced conscious perception, which is directed toward experiencing the external world, and, on the other hand, self-referential mental processes, such as stimulus-independent reasoning and memory retrieval, which are introspective in nature. The common route to narrowing down the mechanism behind conscious processes is to explore the neural correlates of consciousness (NCC), seeking to isolate the distinguishing features of neural activity patterns associated with conscious states (Tononi and Koch, 2008; Aru et al., 2012; Singer, 2015). Mounting evidence suggests that conscious states are related to long-range synchronized brain activity in the beta or gamma frequency band (Crick and Koch, 1990; Desmedt and Tomberg, 1994; Rodriguez et al., 1999; Engel and Singer, 2001; Melloni et al., 2007; Gaillard et al., 2009), with these activity patterns originating from abrupt changes in macroscopic brain dynamics and reflecting the *collective behavior* of large numbers of neurons (Kelso et al., 1992; Freeman, 2004, 2005, 2007). Perception proceeds in rapidly evolving frames with repetition rates lying in the theta frequency band (Freeman, 2004, 2005; Doesburg et al., 2009), while self-referential conscious processes follow the alpha rhythm (Freeman, 2004; Knyazev et al., 2011). In-depth analyses of the empirical data indicate that pattern formation arises from *phase transitions* and that the dynamical characteristics of the brain are due to *criticality* (Kelso et al., 1992; Freeman, 2004, 2005; Kitzbichler et al., 2009; Chialvo, 2010; Tagliazucchi et al., 2012; Plenz et al., 2021).

Consequently, drawing on the available evidence, a natural and reasonable strategy to unveil the mechanism behind conscious processes consists in studying the mechanism underlying critical dynamics. Of particular interest in this regard is *self-organized criticality*, which stands for the ability of a complex system to adjust a control parameter that keeps the system near a critical point of a phase transition (Plenz et al., 2021). Expressed differently, it can be expected that insight into the mechanism used by the brain to control phase transitions will lead to a deeper understanding of the fundamental mechanism by means of which the brain manages to exceed the threshold of consciousness. To pursue this avenue, methods of theoretical physics are required, since the collected empirical data on its own does not reveal the mechanism behind phase transitions. The most promising methods in this context are those of quantum field theory, which have turned out to be remarkably powerful for the description of collective behavior in many-body systems and the explanation of abrupt phase transitions that result in the formation of synchronized neural activity patterns (Del Giudice et al., 1985, 2005; Freeman and Vitiello, 2006, 2008).

Following this line of reasoning, a novel conceptual framework for consciousness based on the foundations of quantum electrodynamics (QED) has been developed (Keppler, 2012, 2013, 2016, 2018, 2020). According to this framework, the key prerequisite that gives rise to the occurrence of phase transitions and the formation of collective dynamics is the interaction of the brain with the ubiquitous vacuum (zero-point) fluctuations of the electromagnetic field, which in the following will be referred to as *zero-point field* (ZPF). This omnipresent field, which is described by a spectrum of normal modes, plays an important role in modern physics and can be interpreted to mean that the vacuum is not a void, but a vibrant sea filled with energy and potentiality (Kuhlmann et al., 2002). Processes involving consciousness are postulated to differ from unconscious processes in that they require *resonant brain-ZPF coupling*, giving fresh impetus to the construction of a theory of consciousness (Keppler and Shani, 2020; Keppler, 2021). In these previous works, however, many questions regarding the details of the coupling mechanism remained unanswered.

Therefore, the purpose of this article is to address the details missing so far and thereby raise the QED-based conceptual framework for consciousness to the maturity level of a solid theory. In concrete terms, the challenges to be tackled in this work can be summarized as follows:

1. Unraveling the brain-ZPF coupling mechanism and providing insight into the principles underlying phase transitions and long-range synchronization.
2. Revealing the distinctive feature of neurophysiological processes accompanied by consciousness, which consists in the *resonant amplification of zero-point modes* as a direct concomitant of the brain-ZPF coupling mechanism.
3. Shedding light on the significance of the ZPF amplification for the formation of conscious states, thus demonstrating the explanatory power of the QED-based theory of consciousness, which, derived from the coupling mechanism, is given the acronym TRAZE.
4. Specifying an experimental design that can be used to test an immediate consequence of the theory, namely, the prediction that normally occurring conscious states are absent under experimental conditions which prevent resonant brain-ZPF coupling.

To address these issues systematically, we take a closer look at cortical microcolumns and employ a field-theoretical model, based on QED, to elucidate their operating principles (Keppler, 2023). Understanding the functioning of microcolumns is essential, as they form the basic functional building blocks of the cortex that sustain advanced cognitive processes. The field-theoretical model will demonstrate that the architecture of the brain is specifically designed to achieve coupling to the ZPF, and it will provide insight into how the brain controls the coupling. Expressed in simplified terms, control takes place via modulation of neurotransmitter concentrations, particularly the concentration of the predominant excitatory neurotransmitter glutamate. It follows from the model that glutamate-ZPF coupling leads to local cortical coherence and, beyond that, causes downstream effects that mediate communication between cortical areas, suggesting that the formation of phase transition-induced, long-range synchronized activity patterns, which according to empirical evidence constitute the NCC, relies on the involvement of the ZPF. These findings support the conclusion that conscious processes are based on the brain’s interaction with the ZPF, underscoring the importance of this omnipresent field for the study of consciousness.

The article is organized in such a way that in Section 2 some important empirical findings on the dynamical characteristics and the architecture of the brain are presented. In Section 3, we will go into the details of the field-theoretical model of cortical microcolumns. Equipped with this theoretical grounding, we then turn to the fundamental mechanisms behind the formation of synchronized neural activity patterns (Section 4) and the postulated mechanism behind the formation of conscious states (Section 5). In Section 6, we address strategies for the empirical corroboration of the postulated mechanism, while Section 7 is dedicated to the concluding discussion and a brief outlook on future research avenues.

## 2 Empirical findings on brain dynamics and brain architecture

### 2.1 Neurotransmitters and criticality

In preparation for the discussion of the field-theoretical model of cortical dynamics, we draw on empirical evidence supporting the decisive role of neurotransmitters in triggering phase transitions. To start with, the propagation of synchronized activity in cortical networks is shown to manifest as neuronal avalanches with sizes and lifetimes obeying power law scaling, which is indicative of a system operating in the critical regime (Beggs and Plenz, 2003; Lombardi et al., 2014; Arviv et al., 2019; Plenz et al., 2021). These avalanches reflect the *collective organization* of cortical activity (Arviv et al., 2019), a key finding being that this organization is driven by the neurotransmitters glutamate and gamma-aminobutyric acid (GABA), as well as the presence of neuromodulators, such as dopamine, serotonin, and acetylcholine (Stewart and Plenz, 2006; Plenz et al., 2021).

The significance of neurotransmitters in initiating phase transitions is further corroborated by studies that relate neurotransmitter concentrations to neurophysiological markers of synchronized brain activity. It is found that there is a correlation between the glutamate concentration and oscillatory power (Gallinat et al., 2006; Lally et al., 2014), that glutamate and GABA control the large-scale synchronization of activity patterns, and that the glutamate levels in cortical and subcortical regions are linked to the functional connectivity between these regions (Duncan et al., 2013). Moreover, computations using phenomenological models aimed at exploring phase transitions in cortical networks emphasize the pivotal role of neurotransmitters by demonstrating that self-organized criticality is regulated by synaptic resources (Levina et al., 2007; Di Santo et al., 2018).

### 2.2 Microcolumns as basic functional units of the cortex

**Figure 1:**
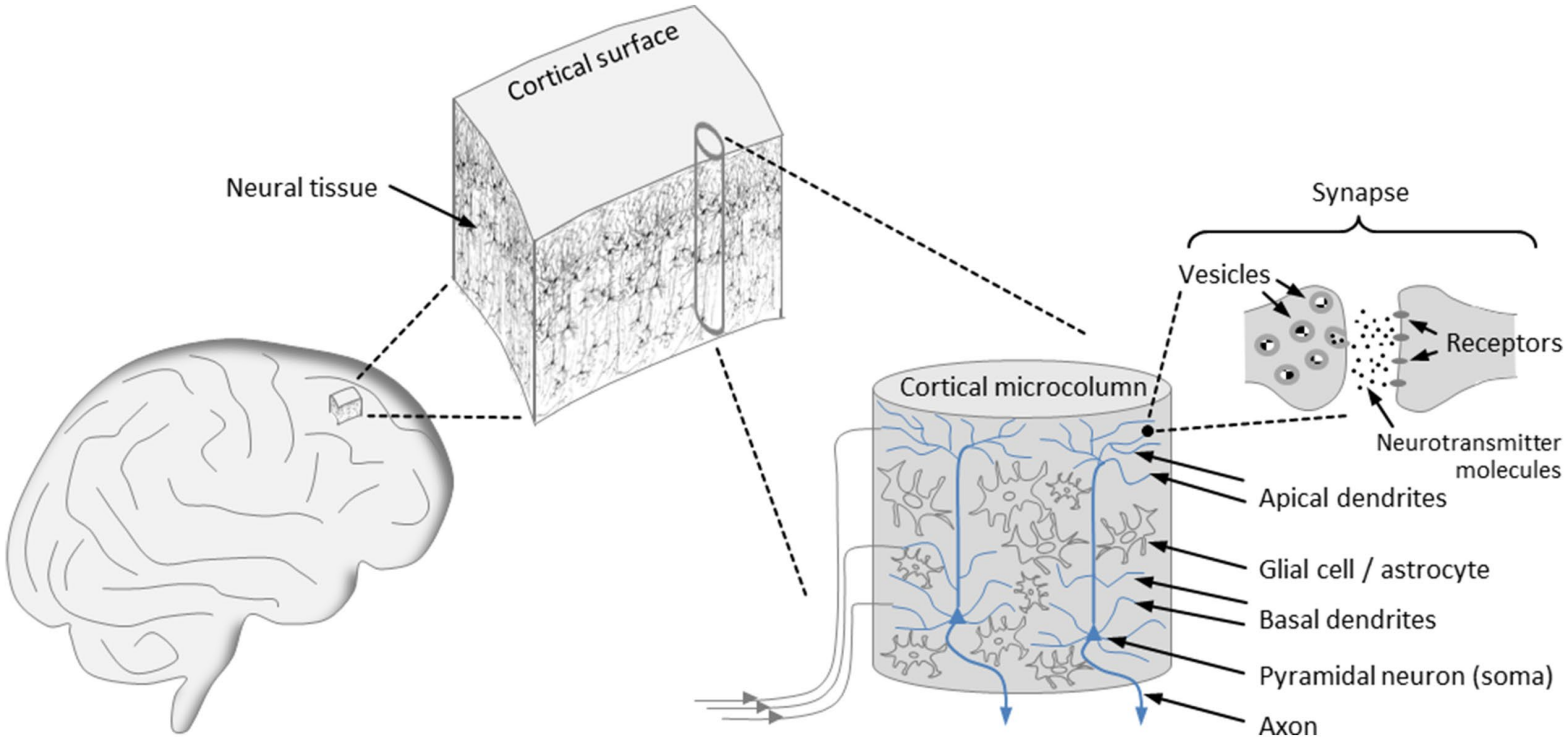
Organization of the cortex. The cortical surface is arranged in horizontal layers and vertical columns, with the microcolumns representing the basic functional units of the cortex that exhibit a uniform architecture. Input signals are transmitted to tens of thousands of synapses that populate the basal and apical dendrites of the pyramidal neurons, which account for the majority of neurons in a microcolumn. At an activated synapse, neurotransmitter molecules mediate signal transmission from the presynaptic to the postsynaptic terminal. The axons of the pyramidal neurons constitute the output channels of a microcolumn, enabling connections to a multitude of other neurons in neighboring or more remote brain areas.

The dynamical characteristics of brain activity hinge not only on molecular components but also on the design principles underlying brain architecture, with our primary focus being on the structural organization of the cortex (see Figure 1). The cortex is arranged in vertical columns and horizontal layers aligned parallel to the cortical surface. For many years, evidence has been accumulating that the minicolumn, also referred to as *microcolumn*, constitutes the *basic functional unit* of the mature cortex (Mountcastle, 1957, 1978, 1997; Buxhoeveden and Casanova, 2002). Although each of the cortical microcolumns is unique in its structural details and its connections to other cortical and subcortical units, their layout is uniform across species, with a typical microcolumn consisting of an estimated 80 to 140 neurons and ranging in diameter from 20 μm to 60 μm (Mountcastle, 1978; Jones, 2000; Buxhoeveden and Casanova, 2002). During evolution, cortical expansion has been accomplished by a continuous increase in the quantity of cortical microcolumns, without altering their size (Mountcastle, 1997).

The microcolumnar architecture of the cortex is well supported by experimental findings pointing to a spatial variation in the optical density of cortical slices at 30–60 μm intervals (Kohn et al., 1997). Notably, bundles of apical dendrites of pyramidal neurons with a diameter of about 30 μm have been identified as repeating structures in cortical tissue (Jones, 2000). In addition, the high degree of neuronal synchronization within individual microcolumns indicates that they constitute a modular system of integrated functional units (Maruoka et al., 2017; Hosoya, 2019).

The microcolumns are grouped into larger assemblies, which themselves form modality-specific areas, such as the visual, the auditory, or the somatosensory cortex. Furthermore, the cortical architecture is characterized by extensive interconnections between the columns, as well as by a high degree of connectivity between the columns and subcortical structures, particularly the thalamus (Mountcastle, 1978, 1997). Fiber bundles emanating from cortical and thalamic modules are directly connected to the dendritic trees of pyramidal neurons, which are essential components of microcolumns and account for about 80% of all neurons (Buxhoeveden and Casanova, 2002). More concretely, thalamocortical and corticocortical fibers transmit input signals to the tens of thousands of excitatory, mostly glutamatergic, synapses that populate the basal and apical dendrites of the pyramidal neurons (Spruston, 2008). The microcolumns also contain interneurons, which are mostly inhibitory and control the activity of pyramidal cells via GABAergic synapses (Buxhoeveden and Casanova, 2002; Spruston, 2008). As it turns out, the periodic triggering of action potentials and the emergence of oscillatory network activity requires the coordination of glutamatergic and GABAergic neurotransmission (Gireesh and Plenz, 2008; Spruston, 2008; Buzsáki and Wang, 2012). An action potential propagates along an axon, which represents the output channel of a pyramidal neuron and enables it to build connections to a multitude of other neurons in neighboring or more remote brain areas (Mountcastle, 1997; Spruston, 2008).

**Figure 2:**
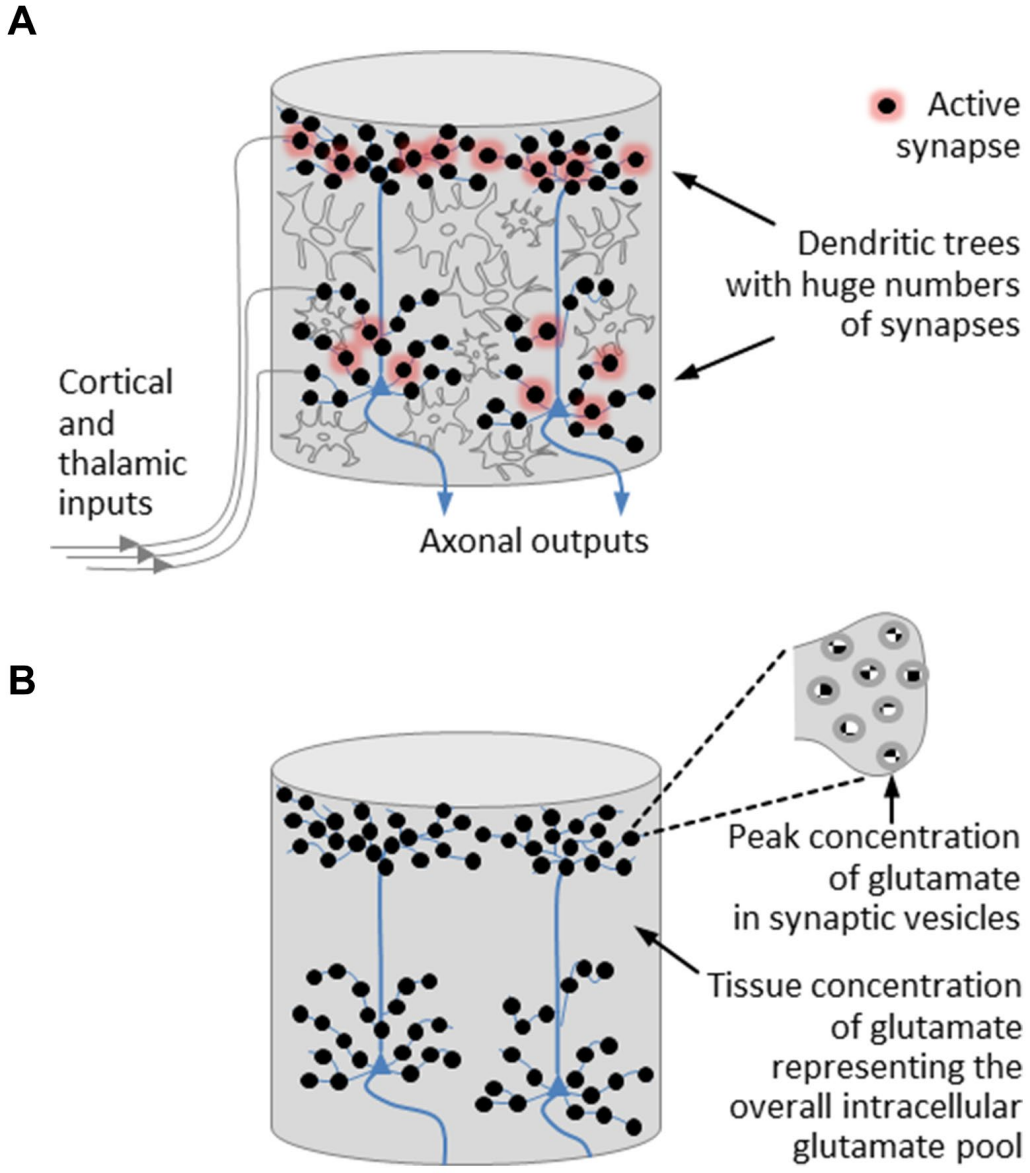
Simplified structural model of a cortical microcolumn. **(A)** Fiber bundles emanating from cortical and thalamic modules are connected to the dendritic trees of the pyramidal neurons, which are densely studded with synapses. In active synapses, neurotransmitter molecules are released from vesicles in which the neurotransmitter concentration reaches its highest level. The most abundant neurotransmitter is glutamate. Regulatory processes that take place in astrocytes stabilize the tissue concentration of glutamate around a mean value. **(B)** A further simplified model of a microcolumn is restricted to pyramidal neurons and the excitatory neurotransmitter glutamate, the presence of which is expressed by two concentrations, namely, a peak concentration encountered in synaptic vesicles and a tissue concentration representing the overall intracellular glutamate pool. On this view, a microcolumn is composed of a bunch of pyramidal neurons enclosed in a glutamate pool.

With regard to the field-theoretical model of a microcolumn, we introduce a few simplifications. On the one hand, we neglect the layered structure of the cortex. On the other hand, we leave interneurons aside and concentrate on pyramidal neurons. In doing so, we disregard components that contribute to the development of oscillatory network activity. However, this will not affect our understanding of the basic operating principles of an individual microcolumn and will not prevent us from gaining insight into the mechanism used by the brain to control phase transitions. The simplified structure of a cortical microcolumn is depicted in Figure 2A.

Let us now revisit those players in the cerebral orchestra that feature prominently in the initiation of phase transitions and the organization of brain activity, namely, the neurotransmitters. Disregarding oscillatory network activity, we can confine ourselves to excitatory neurotransmission. The dominant excitatory neurotransmitter is glutamate, whose level in the brain is several times higher than in any other part of the body and whose concentration in neural tissue is markedly greater than the concentration of any other amino acid (Featherstone, 2010). Peak concentrations of glutamate, as with all other neurotransmitters, are encountered in synaptic vesicles (Scimemi and Beato, 2009; Budisantoso et al., 2013; Wang et al., 2019), while regulatory processes stabilize the tissue concentration of glutamate around a mean value that varies between brain regions (Erecińska and Silver, 1990). These regulatory processes are part of the glutamate-glutamine cycle (Shen et al., 1999; Featherstone, 2010). In more detail, glutamate homeostasis takes place in astrocytes, the most abundant type of glial cells, where metabotropic glutamate receptors control the balance between glutamate uptake and glutamate release (Cartmell and Schoepp, 2000; Meldrum, 2000; Mahmoud et al., 2019).

These insights can be translated into a further simplified structural model of a microcolumn, illustrated in Figure 2B. This model is restricted to pyramidal neurons and the excitatory neurotransmitter glutamate, the presence of which is expressed by two concentrations, namely, a peak concentration encountered in synaptic vesicles and an average tissue concentration that is regulated by glial cells. On this view, *a microcolumn is composed of a bunch of pyramidal neurons enclosed in a glutamate pool*, with the glutamate pool forming a *glutamate-water matrix* due to the high concentration of water in neural tissue (Keppler, 2023).

## 3 Field-theoretical model of a cortical microcolumn

### 3.1 Outline of the model

The preceding considerations pave the road to a field-theoretical functional model of a microcolumn centered on the coupling of the glutamate pool to the ubiquitous vacuum (zero-point) fluctuations of the electromagnetic field, abbreviated as zero-point field (ZPF). The ZPF is a stochastic radiation field that can be decomposed into a spectrum of normal modes, with each normal mode being characterized by a specific frequency. The field-theoretical model is based on the formalism of QED, which has been demonstrated to be perfectly suited for describing the interaction of a many-body system with the ZPF and elucidating the origin of phase transitions (Del Giudice et al., 1985, 2005; Preparata, 1995; Del Giudice and Vitiello, 2006). In what follows, an overview of the essential findings of the QED-based model calculations is given without encumbering the reader with the mathematical formalism that underlies the evolution equations describing the coupled glutamate-ZPF system. Readers interested in the details of the analysis are referred to the original publication (Keppler, 2023). A compact summary of the model calculations can be found in Supplementary Appendix SA.

It turns out from the evolution equations that the *coupling strength of the glutamate pool to the ZPF* is the key parameter governing the dynamical properties of the system. Moreover, the model calculations show that “the dynamical evolution of the coupled matter-ZPF system depends on highly selective resonance conditions which cause one of the molecular excited states to be singled out, subsequently termed preferred excited state, and the evolution of the system to be dominated by those ZPF modes that resonate with this preferred state, subsequently referred to as dominant field modes” (Keppler, 2023). Since electronic excitations are energetically inaccessible and rotational excitations are frozen in the glutamate-water matrix, the preferred excited state of the glutamate molecules is a vibrational excitation.

Studying the early phase of the dynamical evolution, termed *runaway stage*, reveals that the triggering of a phase transition requires the coupling strength to exceed a critical threshold that “depends on the concentration of the molecules and their vibrational excitability” (Keppler, 2023). Such a type of spontaneously occurring phase transition is known as a superradiant phase transition (Hepp and Lieb, 1973; Wang and Hioe, 1973; Del Giudice et al., 1993). Once a phase transition is in progress, “the resonant interaction between the ensemble of molecules and the ZPF drives the entire system toward a stationary state that is characterized by the amplitude of the dominant field modes being significantly boosted and the molecules residing in a collective state” (Keppler, 2023). In other words, “the system undergoes reorganization and switches to a stable configuration in which the molecules and the selected ZPF modes oscillate coherently. This configuration is energetically favored and associated with a decrease in energy per molecule, resulting in the coherent state being shielded by an energy gap” (Keppler, 2023). The establishment of a *coherent state* manifests itself in the formation of a *coherence domain* (Preparata, 1995; Del Giudice and Vitiello, 2006).

Thus, the findings from the solution of the evolution equations can be summarized in such a way that upon exceeding a *critical coupling strength* of the ZPF to the glutamate pool, which amounts to exceeding a *critical concentration of the glutamate molecules*, a phase transition is initiated. Under these conditions, the resonant glutamate-ZPF interaction gives rise to the *amplification of the dominant ZPF modes* and to a dynamical situation in which “the molecules populate a coherent state that can be described as a superposition of the ground state and the preferred excited vibrational state. The coherent state is energetically advantageous and characterized by a reduction of the energy per molecule compared to the non-coherent state” (Keppler, 2023).

**Figure 3:**
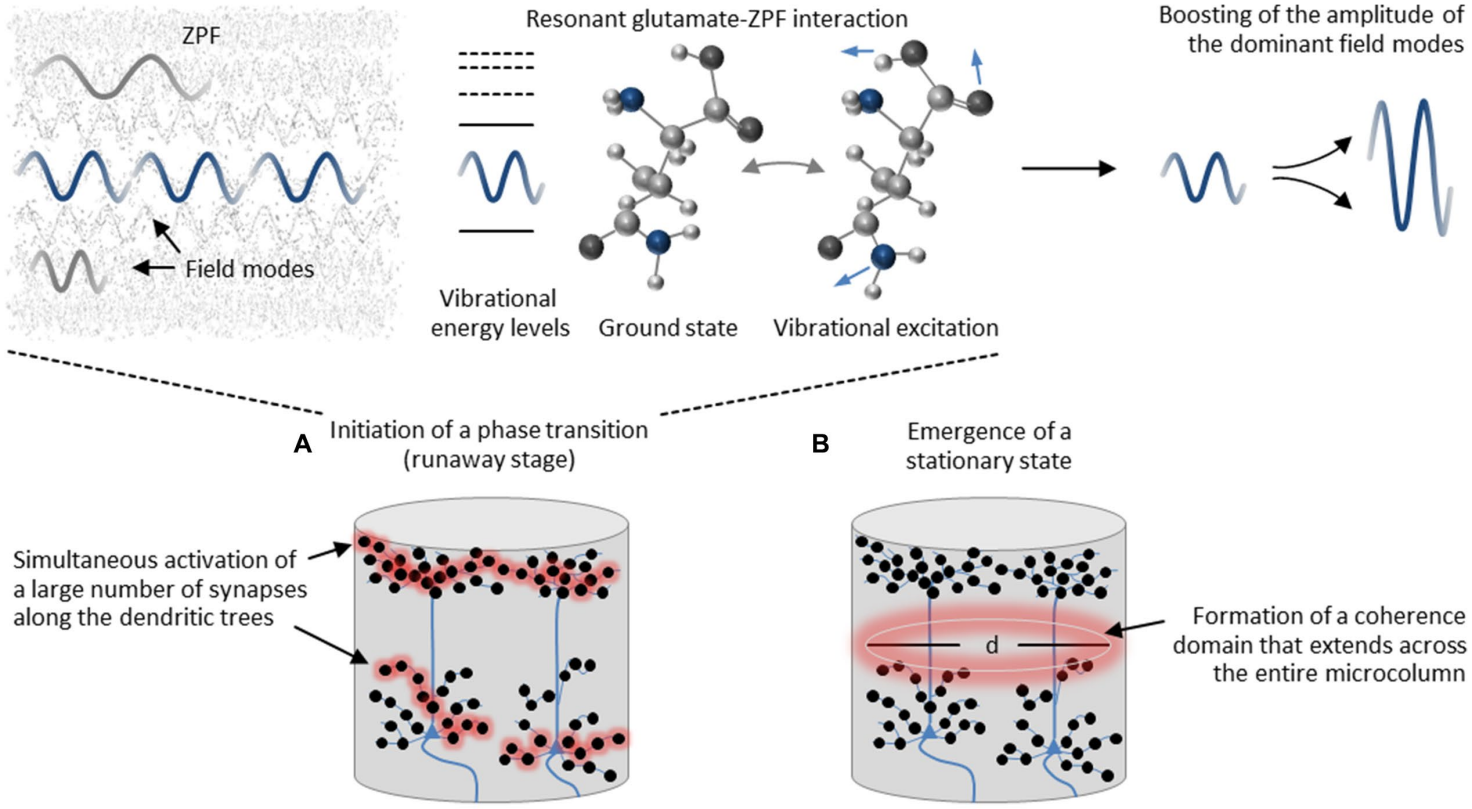
Assumed two-stage process in cortical microcolumns. **(A)** The process starts with the runaway stage that is driven by resonant glutamate-ZPF interaction and results in an amplification of the dominant ZPF modes. The clusters that exhibit suitable conditions for initiating a phase transition are the synaptic vesicles where the peak concentration of glutamate is found. For a phase transition to occur that extends across the entire microcolumn, a large number of synapses along the dendritic trees must be activated simultaneously. **(B)** The phase transition culminates in a stationary coherent state and results in the formation of a coherence domain, the diameter d of which is determined by the wavelength of the dominant ZPF modes.

Proceeding from these model-based insights, the assumed operating principle of a cortical microcolumn depends on a two-stage process. This process starts with the *runaway stage*, see Figure 3A, which is driven by resonant glutamate-ZPF interaction and results in an amplification of the dominant ZPF modes. The clusters that can be expected to meet the requirement for exceeding the critical threshold concentration of glutamate, and thus should exhibit suitable conditions for initiating a phase transition, are the synaptic vesicles where the *peak concentration* of glutamate is found. However, for a phase transition to occur that extends across the entire microcolumn, a large number of synapses must be activated simultaneously along the dendritic trees. More specifically, “the release of highly concentrated glutamate from numerous synaptic vesicles distributed across the dendritic trees generates a single percolation cluster, which is the prerequisite for setting off an avalanche process that drives the glutamate pool within a microcolumn toward a stationary coherent state” (Keppler, 2023). The emergence of a stationary state, see Figure 3B, implicates “the formation of a *coherence domain*, the dynamical properties of which are determined by the *tissue concentration* of glutamate and the diameter d of which is determined by the wavelength of the dominant ZPF modes” (Keppler, 2023). As we will see, the emergence of a coherence domain, which is accompanied by neurophysiological downstream effects, is decisive for understanding the mechanism behind conscious processes.

### 3.2 Viability of the model

To test the viability of the model and demonstrate the plausibility of the proposed operating principle of a microcolumn, quantitative calculations have been performed (Keppler, 2023). The key findings are summarized below. First, it needs to be verified that the peak concentration of glutamate in synaptic vesicles is sufficiently high to induce the runaway stage. Second, it must be shown that at the tissue concentration of glutamate, the resonant glutamate-ZPF interaction arrives at a stationary state, which is essential for the formation of a coherence domain. Third, one has to confirm that the extent of a coherence domain is consistent with the diameter of a microcolumn as derived from empirical data. Fourth, we need to investigate whether a coherence domain is sufficiently protected from disruptive thermal perturbations and thus can withstand rapid decoherence, which is important for eliciting neurophysiological effects. Fifth, we should find indications that the neurophysiological effects associated with the emergence of a coherence domain are crucial for interneuronal communication and the formation of synchronized activity patterns that constitute the NCC. In the following, these five points are dealt with one after the other.

To examine the runaway stage, we need data on the neurotransmitter concentration in synaptic vesicles and on the vibrational modes of glutamate. As for the vesicular glutamate concentration, a realistic value can be assumed to lie in the region of 300 mmol/L (Scimemi and Beato, 2009), with more recent studies even pointing to markedly higher values (Budisantoso et al., 2013; Wang et al., 2019). Concerning the vibrational modes of glutamate, which are in the THz frequency band, it has to be considered that glutamate exists in an aqueous solution in which it is ionized and forms sodium and glutamate ion pairs that are incorporated into the water matrix (Friesen et al., 2021). In the THz frequency range, water exhibits collective vibrations of hydrogen bonds that substantially affect the dynamics of the solutes and can enhance vibrational resonances (Heyden et al., 2010; Mitra and Palit, 2021). This is evident from the measurement of the vibrational resonances of hydrated sodium glutamate, which are considerably increased compared to anhydrous glutamate (Markl et al., 2016). Including these data in the model calculations reveals that in synaptic vesicles the coupling strength of the glutamate-water matrix to the ZPF “*lies in the critical regime*” and that, accordingly, “*the runaway criterion for the initiation of a phase transition*” is satisfied (Keppler, 2023). Moreover, the calculations indicate that the resonance frequency of the preferred excited state of glutamate, and hence the frequency of the dominant ZPF modes that drive the evolution of the system, is 7.8 THz (Keppler, 2023). These findings support the conclusion that a phase transition is induced in the synaptic vesicles. However, as already explained, the initiation of a phase transition that pervades an entire microcolumn necessitates the activation of a large number of closely spaced vesicles, which sheds new light on the arrangement of synapses as observed along the dendritic trees of pyramidal neurons.

After initiating the runaway phase, an avalanche process is ignited that captures the entire glutamate pool of a microcolumn and results in the dynamics of the glutamate pool being governed by resonant coupling to the ZPF. Therefore, we must insert into the evolution equations of the coupled glutamate-ZPF system the tissue concentration of glutamate, the value of which has a lower limit of 8 mmol/L (Pouwels and Frahm, 1998), and in some brain regions reaches values that amount to more than twice the lower limit (Gallinat et al., 2006; Montag et al., 2008). Proceeding from these studies, it is reasonable to assume a value of 12 mmol/L for the average tissue concentration of glutamate, which coincides with the mean glutamate concentration in rodent cortex (Erecińska and Silver, 1990; Hill et al., 2000). The numerical treatment of the evolution equations demonstrates that at this concentration the resonant interaction between the glutamate pool and the ZPF leads to a *stationary solution* where “the amplitude of the dominant field modes is significantly elevated and the system is driven toward a collective state in which the glutamate molecules reside in a superposition of the ground state and the preferred excited vibrational state” (Keppler, 2023).

The establishment of a stationary state is tantamount to the formation of a coherence domain whose diameter d is determined by the wavelength of the dominant ZPF modes, which directly derives from their frequency. A frequency of 7.8 THz yields d ≈ 30 μm, which is “*well in accordance with empirically backed findings* on the extent of a microcolumn” (Keppler, 2023). Notably, this result matches excellently with the finding that bundles of apical dendrites of pyramidal neurons with a diameter of about 30 μm have been identified as repeating structures in cortical tissue (Jones, 2000).

Given the glutamate concentration and the domain size, it follows that about 10^11^ molecules take part in the formation of a coherence domain. Furthermore, the model calculations indicate that in the collective state, in which the molecules of the pool exhibit macroscopic quantum coherence, the energy per molecule is decreased compared to the non-coherent state. Due to the large number of molecules involved, this decrease gives rise to “*an energetically favored state that is shielded by a considerable energy gap*” (Keppler, 2023). The occurrence of such an energy gap is the crucial prerequisite for isolating the collective state from thermal interference and, thus, for preventing rapid decoherence (Del Giudice and Vitiello, 2006), which is confirmed by numerous studies dealing with interacting many-body systems (Mewes et al., 2005; Rey et al., 2008; Poletti et al., 2012; Bouganne et al., 2020). An additional protection of a coherence domain results from the presence of water and the properties of the glutamate-water matrix. Since under the boundary conditions encountered in biological tissue, water itself is found to be composed of coherence regions (Del Giudice et al., 2010, 2013), disruptive thermal perturbations are strongly suppressed within the domain and can only attack via its surface (Del Giudice et al., 2005). Consequently, we have an overall situation in which “all the molecules of the glutamate-water matrix oscillate in unison with the dominant ZPF modes” and only a very small fraction of the molecules that constitute a coherence domain are vulnerable to the influx of thermal energy from the environment, suggesting that “*under the special conditions encountered in a cortical microcolumn, the formation and temporary maintenance of macroscopic quantum coherence is very plausible*” (Keppler, 2023). The model calculations thus invalidate the frequently put forward argument that quantum phenomena cannot occur under the wet and warm conditions of the brain (Tegmark, 2000; Koch and Hepp, 2006).

The emergence of a coherence domain induces two types of downstream effects. On the one hand, there are effects that can be attributed to the coherent state of the glutamate molecules. On the other hand, effects occur that originate from the amplification of the dominant ZPF modes.

Let us turn to the first category of effects. As mentioned earlier, the coherent state of the glutamate molecules is described as a superposition of the ground state and the preferred excited vibrational state. Due to the vibrational excitation of the glutamate molecules, vibrational energy can be imparted to the glutamate receptors, causing conformational changes of the receptors and initiating the opening of ion channels, which coincides with the principle of receptor activation through agonist-specific vibrational energy transfer (Kubo et al., 2001, 2003). This implies that the coherent state of the glutamate pool gives rise to “*enhanced synaptic signal transduction*” (Keppler, 2023).

As for the second category of effects, we noted previously that the amplitude of the dominant field modes, i.e., those ZPF modes that are strongly coupled to the glutamate molecules, is considerably elevated. In addition, the model calculations also show that the formation of the stationary state of the coupled glutamate-ZPF system entails a frequency shift of the dominant ZPF modes from 7.8 THz to 30 GHz, which lies in the microwave frequency range and means that an intracolumnar microwave radiation field is generated as a result of the resonant glutamate-ZPF interaction (Keppler, 2023). Both theoretical studies and experimental findings indicate that microwaves induce collective excitations in membranes and modulate ion flows across membranes by regulating voltage-gated ion channels (Pickard and Rosenbaum, 1978; Bond and Wyeth, 1986; Beneduci et al., 2012; Li et al., 2014). In particular, studies demonstrate that microwaves interact directly with voltage-gated ion-channels in the plasma membrane of pyramidal neurons, thereby increasing the membrane permeability, facilitating the electrical signal propagation, and affecting the shape of action potentials (Pikov et al., 2010; D’Agostino et al., 2018). These insights suggest that “*the intracolumnar microwave radiation field plays the role of modulating voltage-gated ion channels and controlling axonal signal transduction*” (Keppler, 2023).

We can conclude that quantitative model calculations corroborate the proposed operating principle of microcolumns. This principle can be formulated in such a way that “the functioning of microcolumns is based on resonant glutamate-ZPF interaction and resultant macroscopic quantum coherence, which produces two types of downstream effects in pyramidal neurons. These are the enhancement of synaptic signal transduction and the regulation of axonal signal transduction” (Keppler, 2023). It is reasonable to assume that both effects are crucial for interneuronal communication and synchronization.

Proceeding from this in-depth analysis, it is to be expected that all pyramidal neurons located in the coherence domain of a microcolumn, defining the zone of influence of the intracolumnar microwave radiation field, display a high degree of synchronized activity, which is confirmed experimentally (Maruoka et al., 2017; Hosoya, 2019). Furthermore, due to the simultaneous effect of the resonant glutamate-ZPF interaction on synaptic and axonal signaling, dendritic and somatic activity should be highly correlated in individual pyramidal neurons, which is supported by empirical data (Beaulieu-Laroche et al., 2019).

## 4 Mechanisms behind the formation of synchronized neural activity patterns

The model-based insights into the functional principle of microcolumns shed new light on the neural mechanisms at work in conscious processes. These insights suggest that the communication between neurons, and thus the formation of synchronized neural activity patterns constituting the NCC, is governed by resonant glutamate-ZPF coupling. More specifically, the model indicates that “*long-range synchronization in the brain emerges through a bottom-up orchestration process involving the ZPF, a key characteristic of this process being the formation, propagation, and synchronization of coherence domains*” (Keppler, 2023). This dynamical process encompasses all levels of brain organization: the glutamate-ZPF interaction takes place at the microscopic level, leading to the establishment of coherence domains at the mesoscopic level, where coherence-triggered downstream effects occur that regulate the macroscopic behavior of the system. This cascade lies at the heart of a coherence-based model of cortical dynamics.

The pyramidal neurons in the cortical microcolumns are ideally suited to control the glutamate-ZPF interaction and thus the entire functional chain. As outlined in Section 3.1, control can be achieved by varying the proportion of simultaneously activated synapses distributed across the dendritic trees. The decisive point here is that glutamate release from a minimum number of synaptic vesicles is required to initiate a phase transition that drives the entire glutamate pool of a microcolumn toward a coherent state. This explains the important role of the thalamus, including neuromodulatory brainstem inputs to the thalamus, for waking consciousness (Modolo et al., 2020), which is due to the fact that, in addition to corticocortical inputs, thalamocortical fibers project to tens of thousands of glutamatergic synapses that populate the dendrites of pyramidal neurons (Spruston, 2008). It is only through the large number of synapses triggered by the thalamus that the critical threshold of activated synapses necessary for the induction of a microcolumnar phase transition and the formation of a coherence domain can be exceeded.

The significant contribution of pyramidal neurons to the cellular mechanisms underlying conscious processes is also highlighted in other works. In concrete terms, it is proposed that pyramidal neurons “act as gates that control the evolution of global activity patterns” and that conscious states rely on a “gating mechanism that regulates the propagation of activity patterns in the thalamocortical system” (Aru et al., 2020), with this mechanism being disrupted in unconscious states, such as during anesthesia. More precisely, it is assumed that the apical dendrites of pyramidal neurons serve as switches (Aru et al., 2020), and that perceptual inputs that flow into the basal compartments of pyramidal neurons only transcend the threshold of consciousness if apical amplification processes are turned on (Larkum, 2013; Marvan et al., 2021). This gives rise to the notion that apical amplification, which necessitates continuous thalamocortical and corticocortical feedback, enables the context-dependent selection of perceptual stimuli and their integration into a conscious perceptual experience (Marvan et al., 2021).

**Figure 4:**
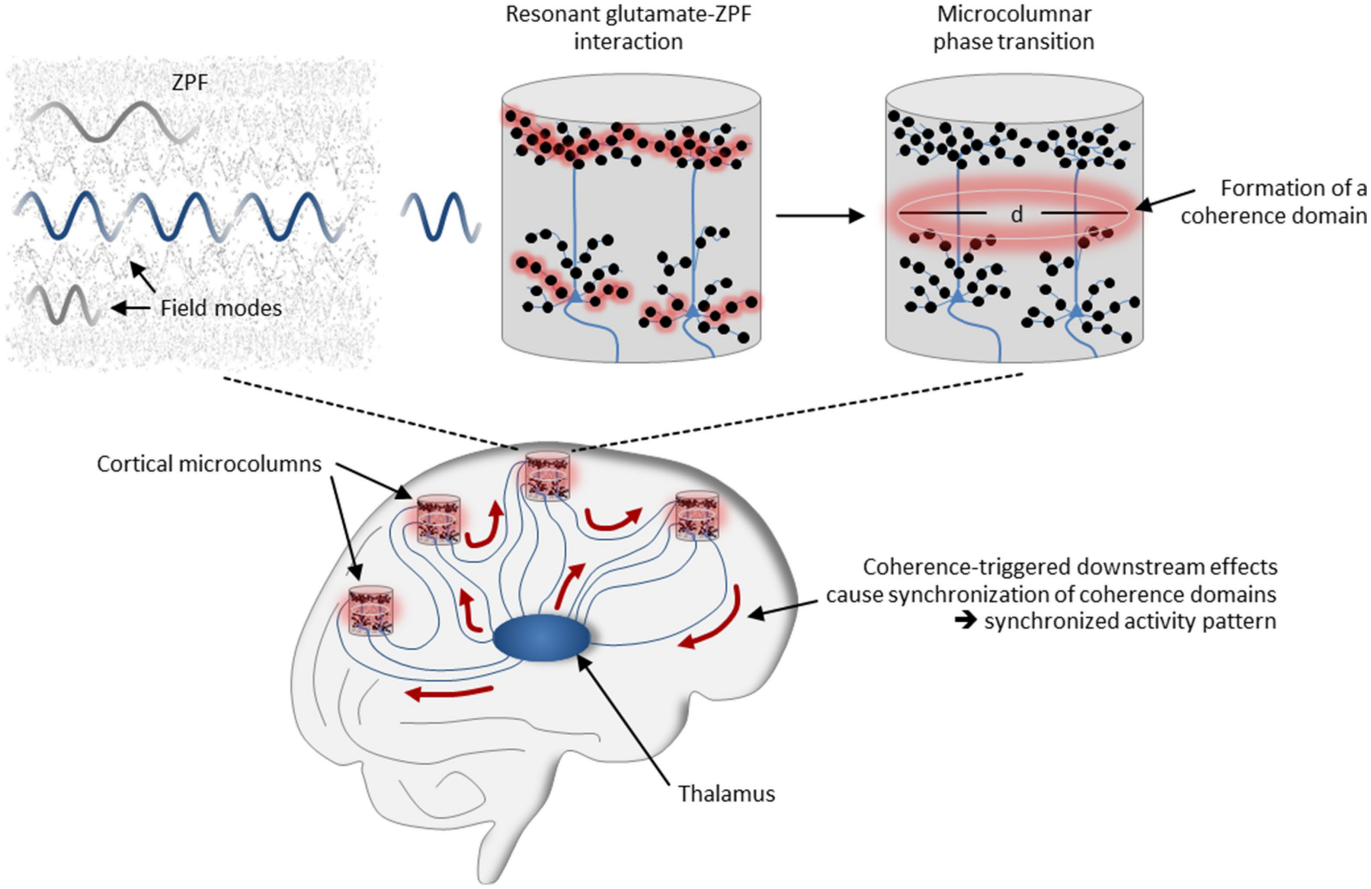
Mechanisms underlying the formation of synchronized neural activity patterns. The field-theoretical model of cortical dynamics suggests that long-range synchronization is the outcome of a ZPF-driven orchestration process involving thalamocortical and corticocortical loops. In those microcolumns in which the number of activated synapses exceeds a critical threshold, resonant glutamate-ZPF coupling sets in, resulting in microcolumnar phase transitions and the formation of coherence domains. Coherence-triggered downstream effects cause synchronization of the coherence domains and produce a synchronized activity pattern.

These neural mechanisms that have been identified as essential for conscious perception can now be reinterpreted using our coherence-based model of cortical dynamics. According to this interpretation, sensory inputs are routed to the microcolumns of the corresponding modality-specific cortical regions, where they terminate on the basal dendrites of pyramidal neurons. Via corticocortical and thalamocortical loops, signals are fed back to the sensorially stimulated microcolumns, in this case to the apical dendrites of the pyramidal neurons. In those microcolumns in which the number of activated synapses exceeds a critical threshold, resonant glutamate-ZPF coupling sets in, resulting in microcolumnar phase transitions and the formation of coherence domains. Coherence-triggered downstream effects cause synchronization of the coherence domains and produce a synchronized activity pattern that involves those microcolumns in which a phase transition takes place. The mechanism is illustrated in Figure 4. Each synchronized activity pattern is thus characterized by a specific assembly of activated microcolumns in which the criticality criterion is fulfilled, meaning that these microcolumn assemblies are bound together by critical dynamics. Proceeding from the evidence that conscious states are related to long-range synchronized activity patterns whose formation arises from phase transitions (see Section 1), our model of cortical dynamics therefore suggests a *sharper specification of the NCC*, such that these are *microcolumn assemblies that are coherently bound together by ZPF-mediated critical dynamics*.

Revealing these mechanisms provides deep insight into the dynamical characteristics of brain activity found in the context of conscious processes. However, these mechanisms do not yet offer a conclusive explanation for consciousness, i.e., for the fact that the neural processes described above are accompanied by phenomenal properties. In order to understand the fundamental mechanism underlying the emergence of conscious states, we must return to the finding that the formation of synchronized neural activity patterns is governed by resonant brain-ZPF coupling. The crucial point for our further considerations is that the resonant interaction between the brain and the ZPF gives rise to the *amplification of specific ZPF modes*, namely, those modes that play a dominant role in the interaction. This leads us to conclude that the ZPF is the key to the understanding of consciousness and that *the distinctive feature of neurophysiological processes associated with consciousness consists in the modification of the ZPF*. In the following section, we will explore this path in greater detail.

## 5 Postulated mechanism behind the formation of conscious states

The important role of the ZPF becomes apparent by looking at the worldview of modern physics, as reflected in the standard model of particle physics (Cottingham and Greenwood, 2007). In the standard model, all the particles and fundamental forces are described by quantum field theories whose self-consistent formulation presupposes the existence of an ever-present ocean of energy. Furthermore, approaches addressing the foundations of quantum physics have shown that the ZPF is the root cause of quantum phenomena (De la Peña and Cetto, 1994, 1995; De la Peña et al., 2015; Cetto and de la Peña, 2022). Against this background, it is not surprising that the ZPF drives macroscopic quantum coherence in the brain and controls neurophysiological processes.

Considering the prominent position of the ZPF in the construction plan of the universe, it seems reasonable to conceive of the ZPF as the fundamental substrate of consciousness (Keppler and Shani, 2020; Keppler, 2021). This idea can be translated into the postulate that the *ZPF is inherently sentient*, or in other words, that the universe is permeated by a ubiquitous psychophysical field that reveals itself extrinsically through its energetic properties, while intrinsically it is of a phenomenological nature (Keppler and Shani, 2020), suggesting that *the spectrum of phenomenal qualities is represented by the vibrational spectrum of the ZPF* and that “each normal mode is associated with an elementary shade of consciousness” (Keppler, 2021). From this dual-aspect perspective, the omnipresent ZPF “thus constitutes an entity that embodies the principles of physics and at the same time contains within itself the phenomenological basis of ultimate reality” (Keppler and Shani, 2020).

**Figure 5:**
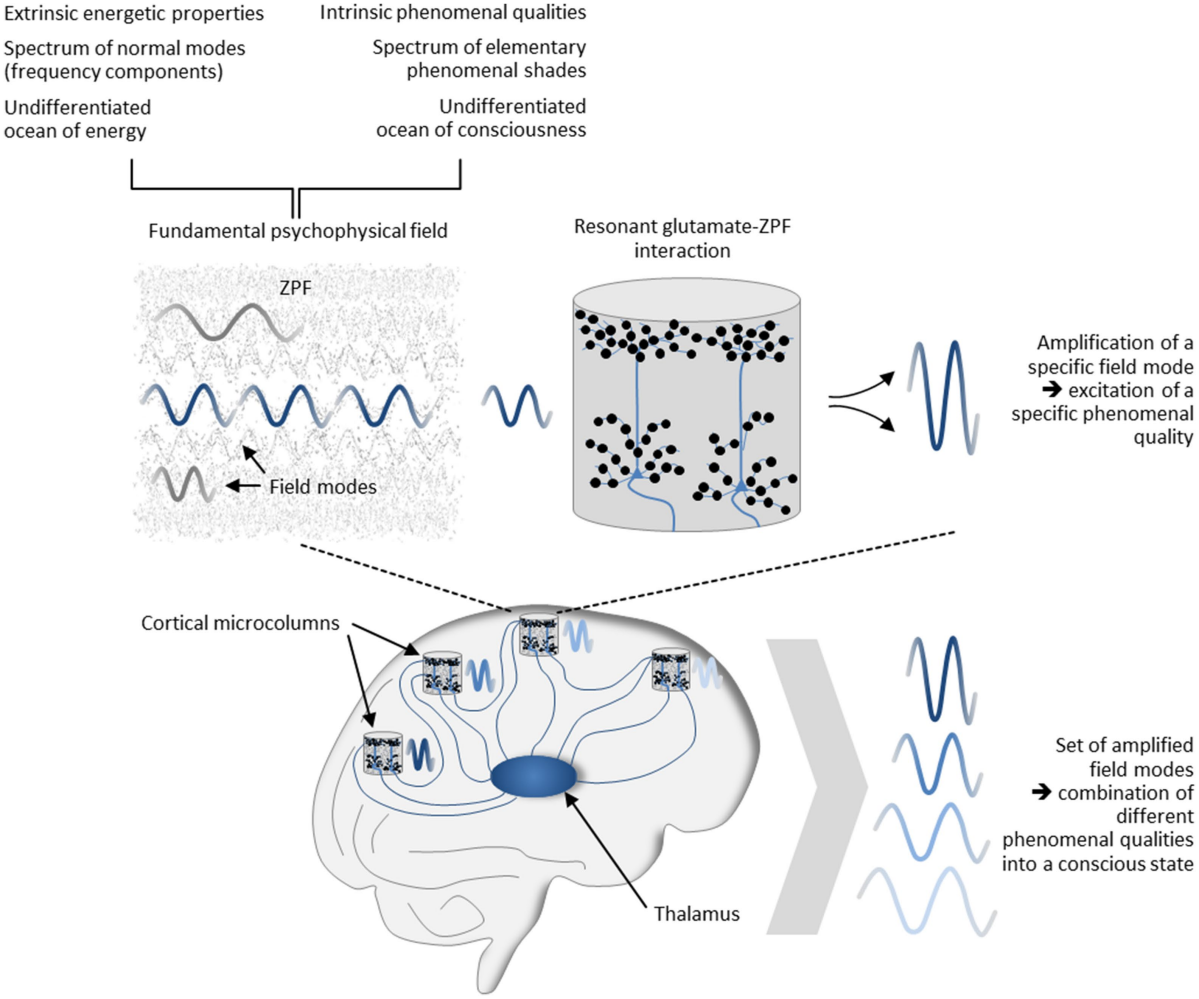
Mechanism underlying the formation of conscious states. Postulating that the ZPF is a fundamental psychophysical field exhibiting extrinsic energetic properties and intrinsic phenomenal qualities, and assuming that the spectrum of phenomenal qualities is represented by the vibrational spectrum of the ZPF, the significance of resonant brain-ZPF interaction for the emergence of conscious states is apparent. The interaction results in the amplification of specific ZPF modes, which goes hand in hand with the excitation of specific phenomenal qualities. Accordingly, the formation of a set of amplified field modes can be construed as the combination of different phenomenal qualities into a conscious state. The set of phenomenal qualities that are merged into a conscious state is determined by those microcolumns that undergo a phase transition and are involved in the formation of a synchronized neural activity pattern.

The psychophysical field hypothesis now allows some inferences to be drawn. Since in the stochastic ground state of the ZPF no modes are amplified and singled out from other modes, the unmodified ZPF can be understood as an undifferentiated ocean of consciousness “that carries an enormous range of potentially available phenomenal nuances” (Shani and Keppler, 2018). This clearly implies that the distinctive feature of conscious systems must consist in their capacity to modify the ZPF, meaning that “the formation of concrete conscious states is confined to those systems that can dynamically interact with the ZPF” (Keppler, 2021). This insight casts new light on the mechanism underlying conscious systems and explains the significance of resonant brain-ZPF interaction for the emergence of conscious states. The key characteristic of this type of interaction is the amplification of specific ZPF modes, which in the dual-aspect picture of the ZPF is inextricably linked with the *excitation of specific phenomenal qualities*. In other words, resonant brain-ZPF coupling results in a set of amplified field modes, which according to the psychophysical field hypothesis can be construed as the combination of different phenomenal qualities into a conscious state. The set of phenomenal qualities that are merged into a conscious state is determined by those microcolumns that undergo a phase transition and are involved in the formation of a synchronized neural activity pattern. The mechanism is depicted in Figure 5.

At this point, it should be emphasized that even though resonant glutamate-ZPF interaction is always initiated via the base frequency of 7.8 THz, the frequency of the amplified field modes is in fact microcolumn-specific. As noted previously, this is because the formation of a stationary state of the coupled glutamate-ZPF system entails a shift of the base frequency to an oscillatory frequency that lies in the microwave frequency range, with the specific shift factor being dependent on the tissue concentration of glutamate (Keppler, 2023). This leads to the conclusion that, following the dual-aspect notion of the ZPF, the phenomenal quality associated with a microcolumn is controlled by the local glutamate concentration, which varies between brain regions (Pouwels and Frahm, 1998).

The processes described above lend explanatory power to the QED-based theory of consciousness, which is denoted by the acronym TRAZE in reference to the *resonant amplification of zero-point modes*. While conventional materialist approaches attribute the emergence of phenomenal states to a mysterious mechanism whose operating principle remains obscure (Levine, 1983; Chalmers, 1995; Nagel, 2012), TRAZE provides a transparent and comprehensible mechanism for the formation of conscious states. The explanatory power of the theory is due to a paradigm shift according to which consciousness is not regarded as a phenomenon that arises from insentient system components, but recognized as the inherent nature of the omnipresent ZPF that can be accessed under suitable conditions. Based on the presented mechanism, access is restricted to those systems that can couple resonantly to the ZPF and have the capacity to modify the undifferentiated ground state of the ZPF (Keppler, 2021). Consequently, *the formation of conscious states requires macroscopic quantum coherence*, as this is the appropriate form of organization that leads to a modification (more precisely, a selective amplification) of the ZPF.

To conclude this section, the distinction between the neural correlate of a conscious state and the seat of consciousness deserves to be explicitly highlighted. The central aspect of the QED-based model behind TRAZE is the dynamic interplay between the ZPF and the brain, or more precisely, the resonant interaction between ZPF modes and the glutamate pool of the microcolumns. On the part of the brain, resonant glutamate-ZPF interaction results in the formation of long-range synchronized activity patterns, involving those microcolumns in which a phase transition takes place. Therefore, according to TRAZE, the neural correlate of a conscious state is an assembly of microcolumns that are coherently bound together by critical dynamics, with the ZPF playing the role of the orchestrator of the collective system behavior (see Section 4). On the part of the ZPF, resonant glutamate-ZPF coupling gives rise to a set of amplified, phase-locked field modes whose selection depends on the glutamate concentrations of those microcolumns that are dynamically fused into an assembly. In TRAZE, the ubiquitous ZPF is postulated to be the fundamental, intrinsically phenomenal substrate of consciousness, with each field mode being assigned an elementary phenomenal quality. This implies that the ZPF is the ultimate seat of a conscious state whose phenomenological profile is determined by an assembly-specific combination of field modes (see Figure 5). Crucially, the integrative nature of the brain-ZPF coupling mechanism (based on macroscopic quantum coherence) accounts for the amalgamation of elementary phenomenal qualities into a multi-faceted, unified conscious percept. Provided they are part of a ZPF-orchestrated assembly, contributions from very different modality-specific brain areas (visual, auditory, somatosensory, gustatory, olfactory, emotional) can in this way be integrated into a complex state of consciousness. These insights are consistent with other studies that emphasize the strengths of field theories of consciousness in explaining phenomenal binding (Hunt and Jones, 2023).

## 6 Empirical corroboration of the postulated mechanism

Two strategies are conceivable to substantiate the existence of the hypothesized mechanism behind the formation of conscious states. The first route pursues the goal of providing direct or indirect evidence for resonant brain-ZPF coupling during conscious states, while the second route seeks to demonstrate that the disruption of the coupling mechanism results in the absence of conscious states.

Direct evidence for the coupling mechanism can be provided by verifying the existence of macroscopic quantum coherence in cortical microcolumns. Such verification can be achieved by determining robust quantum indicators, such as information about the population of glutamate states, which do not require complex non-invasive measurements and are suited for the detection of quantum dynamics in biological systems (Li et al., 2012). To provide indirect empirical evidence for the coupling mechanism, one can exploit the finding that the coherent state of the glutamate molecules is associated with a decrease in energy, which is why a phase transition in a cortical microcolumn should be attended by a collective emission of photons (Keppler, 2020, 2021). This effect, known as biophoton emission, can be demonstrated experimentally using sensitive measuring methods (Popp et al., 1994; Cohen and Popp, 1997; Popp, 2003). Interestingly, glutamate-induced biophotonic activity has been detected in the mouse brain, which sets in upon exceeding a critical glutamate concentration (Tang and Dai, 2014).

It seems natural to demonstrate the postulated coupling mechanism, which relies on a base frequency of 7.8 THz, using brain stimulation studies. However, THz radiation in the frequency range between 1 and 10 THz interacts strongly with water molecules, which limits its penetration depth into biological tissue to several hundred micrometers. As a result, external THz radiation cannot penetrate the skin and reach the inside of the skull (Nikitkina et al., 2021), making it impossible to experimentally substantiate the mechanism using conventional brain stimulation methods. The essence of the mechanism is to utilize the local fluctuations of the electromagnetic field within a microcolumn, which requires novel experimental strategies.

Let us therefore focus on the experimental strategy that aims to prevent the coupling of the brain to the ZPF. A prediction ensuing from TRAZE is that conscious states cannot occur when the brain-ZPF coupling is inhibited and there is no modification of the ZPF, the presumed substrate of consciousness. This prediction stands in clear contrast to the conventional physicalist position, which holds that consciousness originates from neurophysiological processes taking place in the brain. To provide clarity, the question has to be answered whether, under experimental conditions that disrupt the coupling of the ZPF to brain areas chosen for the test, the phenomenal states normally to be expected do not arise. An affirmative test result would demonstrate that phenomenal qualities are not emergent properties of the brain. In the following, an experimental design is described by which the local structure of the field can be manipulated in such a way that resonant glutamate-ZPF coupling in cortical microcolumns is prevented. First-person accounts can be used to validate the expected absence of conscious experiences.

The starting point of the model-based test strategy is the finding that, as outlined in Section 3.2, the frequency of the ZPF modes that dominate the evolution of the coupled glutamate-ZPF system is 7.8 THz. Accordingly, the ZPF modes that occupy a narrow frequency band around 7.8 THz are essential for the integrity of the coupling mechanism. The central idea behind the test is to influence the structure of the ZPF so that those ZPF modes that lie in the relevant frequency band are selectively eliminated. Such a manipulation can be performed locally in a small array of microcolumns. By excluding the relevant ZPF modes, resonant coupling of the glutamate pool to the ZPF cannot establish, causing the functional breakdown of the affected microcolumns. The prediction is that this breakdown entails the absence of phenomenal states usually experienced. It is crucial to note that in this test scenario only the local structure of the ZPF is manipulated, without making any changes to the brain. In this way, the design of the experiment is specifically tailored to demonstrate that phenomenal awareness is a phenomenon that does not emerge from the brain.

**Figure 6:**
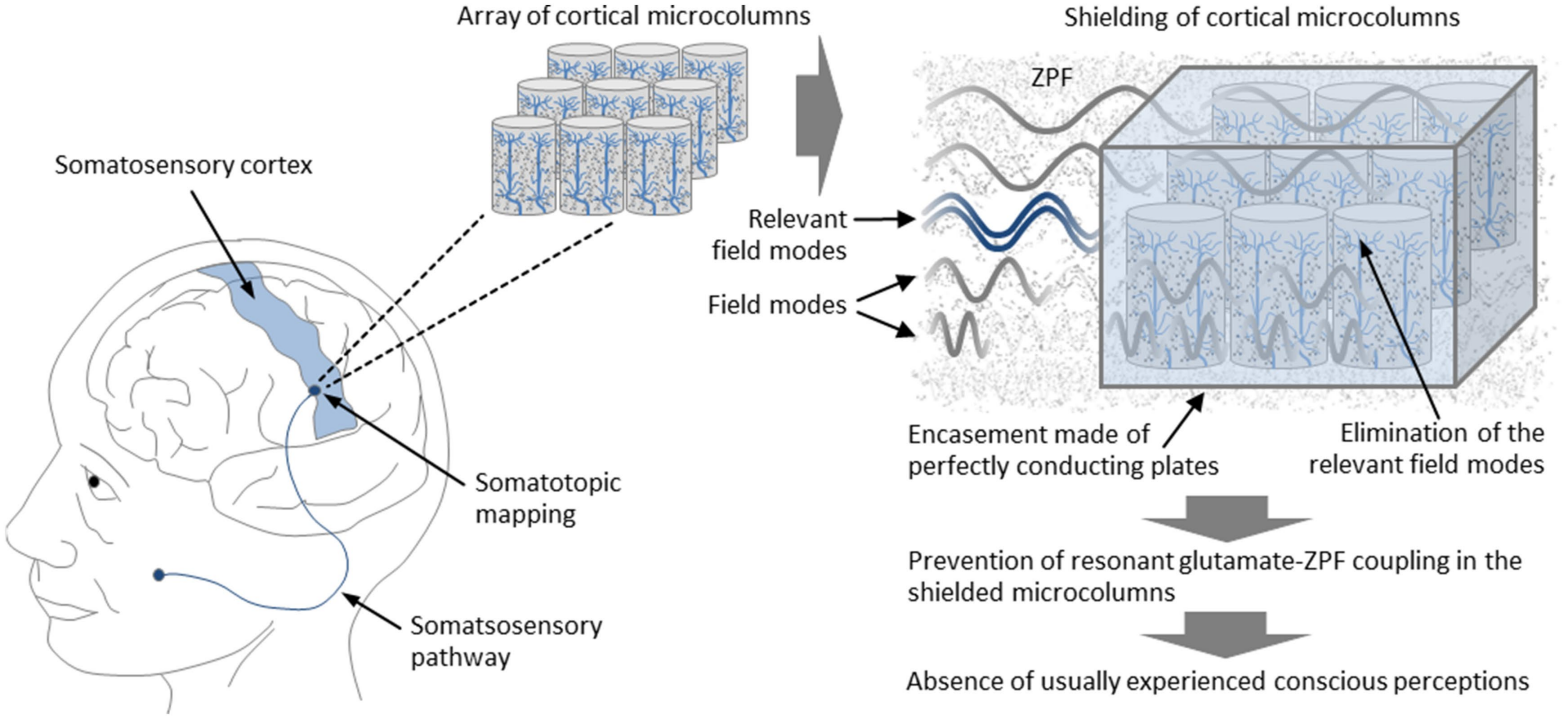
Suppression of conscious perceptions through targeted manipulation of the ZPF. It is proposed to choose an array of microcolumns from the somatosensory cortex, which is organized strictly somatotopically, meaning that there is an unambiguous mapping between body regions and cortical regions. The elimination of the relevant ZPF modes that play a dominant role in the glutamate-ZPF interaction can be achieved by encasing the array with thin, perfectly conducting plates, preventing resonant glutamate-ZPF coupling in the shielded microcolumns. According to the theory to be tested, the inhibition of resonant glutamate-ZPF coupling disrupts the functional principle of the affected microcolumns, so that the usually experienced phenomenal states are expected to be absent. This should be demonstrable by means of first-person accounts of the test subjects. In concrete terms, it is predicted that despite stimulation of the body region associated with the shielded microcolumns, the characteristic conscious perceptions (experience of pain, perception of temperature, sensation of touch) do not occur.

To further specify the experimental setup, it is proposed to choose an array of microcolumns from the somatosensory cortex. This part of the cortex is organized strictly somatotopically, meaning that there is an unambiguous mapping of a body region to the chosen cortical region (Sanchez Panchuelo et al., 2018). Particularly suitable are cortical areas that are associated with highly sensitive regions of the body, such as regions on the hands, on the feet, or in the face. To properly manipulate the local structure of the ZPF in the chosen array of microcolumns, one has to eliminate the ZPF modes that lie in the narrow frequency band around 7.8 THz, which can be achieved by encasing the array with thin, perfectly conducting plates. This type of configuration, which corresponds to configurations used in measurements and technological applications of the Casimir effect (Lamoreaux, 2005; Stange et al., 2021), imposes boundary conditions on the ZPF such that in the space between the plates only ZPF modes are allowed whose wavelengths are integer fractions of twice the plate spacing. Consequently, certain frequency bands can be eliminated by adjusting the plate spacing appropriately. Taking, for example, a 3-by-3 array of microcolumns, the plate spacing is approximately 90 μm, implying that the set of allowed frequencies comprises only integer multiples of 1.67 THz. Such a configuration effectively excludes the frequency band most relevant to the glutamate-ZPF interaction, preventing resonant glutamate-ZPF coupling in the shielded microcolumns. According to the theory to be tested, the inhibition of resonant glutamate-ZPF coupling disrupts the functional principle of the affected microcolumns, so that the usually experienced phenomenal states are expected to be absent. This should be demonstrable by means of first-person accounts of the test subjects. In concrete terms, it is predicted that despite stimulation of the body region associated with the shielded microcolumns, the characteristic conscious perceptions (experience of pain, perception of temperature, sensation of touch) are suppressed. The setup of the experiment is illustrated in Figure 6.

Of course, all ethical guidelines must be complied with when conducting this experiment. For instance, test subjects may be considered where a hole in the skull provides direct access to the chosen cortical region. Alternatively, the test can be performed using rodents. In this case, however, the animals’ perceptions (more precisely, the absence of perceptions) must be inferred from their behavior. The major challenge in conducting the experiment is to ensure that the neuronal connections are not damaged by inserting the plates into the cortical tissue. It is obvious that such technical aspects and many more details need to be clarified in preparation for the test, which is why the experimental setup presented here should be understood as a conceptual draft.

## 7 Discussion and outlook on future research avenues

Having established the foundations of the theory, let us look at the positioning of TRAZE in relation to other theories of consciousness. The aim here is not to perform an all-encompassing review of the theory landscape. Rather, in the following discussion we will restrict ourselves to the most prevalent theories that have conceptual intersections with TRAZE. These theories can be divided into three classes, namely, theories that rely on a neural substrate of consciousness (class 1), classical electromagnetic theories of consciousness (class 2), and quantum theories of consciousness (class 3).

Representatives of class 1 attribute consciousness to complex dynamic interactions between neurons, with different branches of this class emphasizing different aspects of the dynamic interactions. One branch views the generation of activity patterns exceeding the threshold of consciousness as a collective phenomenon and underlines the necessity of a *global workspace* that coordinates the activity of a large number of neurons distributed across functionally specialized brain regions (Baars, 1988, 2005; Dehaene and Naccache, 2001; Dehaene et al., 2006). A second branch highlights the importance of the thalamocortical system, which is assumed to operate as a *dynamic core* that is capable of forming an enormous range of differentiated, transiently stable states (Tononi and Edelman, 1998; Edelman, 2003; Seth et al., 2006). A third branch agrees with the notion of consciousness being associated with the formation of transient coalitions of neurons but underscores the significance of *synchronization* among brain areas for the binding of separate features into a unified conscious percept (Crick and Koch, 1990, 2003). Yet another branch stresses the necessity of *recurrent processing* for the generation of conscious states (Lamme, 2006). Apart from their different emphases, all these theories share a common denominator, namely, the basic premise that conscious experiences emerge from or are identical with neural activity patterns, meaning that qualia are assumed to be represented by active cell assemblies. It is precisely this reduction of consciousness to a neural substrate that leads to explanatory gaps (Levine, 1983; Chalmers, 1995; Nagel, 2012), the most evident of which is the lack of unambiguous, plausible differentiators between conscious and unconscious processes.

The strength of TRAZE is that it unifies all the aforementioned class 1 theories at a more fundamental level by offering a universal mechanism that not only incorporates all the dynamical characteristics outlined above, but also provides a conclusive explanation for the formation of phenomenal states, thereby drawing an unambiguous, plausible dividing line between conscious and unconscious processes. A central element of this mechanism is the resonant coupling of the brain to the omnipresent ZPF that acts as a hidden coordinator of brain activity and can be understood as a global workspace in the truest sense of the term. The dynamic brain-ZPF interaction induces the formation, propagation, and synchronization of coherence domains, resulting in the emergence of synchronized activity patterns. As we have seen, this process is based on a phase transition in cortical microcolumns, the triggering of which requires recurrent processing in the form of corticocortical and thalamocortical feedback in order to exceed a critical threshold of activated synapses. The crucial point is that the dynamic interplay between the brain and the ZPF gives rise to the amplification of specific ZPF modes, which under the assumption of the psychophysical dual-aspect nature of the ZPF goes hand in hand with the excitation of specific phenomenal qualities. Therefore, the initiation of a phase transition and the establishment of macroscopic quantum coherence, accompanied by a modification of the ZPF ground state, is a necessary condition for the emergence of a conscious state.

These insights place the ZPF at the center of a fundamental theory of consciousness and expose this ubiquitous field as the connecting link to the fundamental theories of physics. From this perspective, the neural activity patterns constituting the NCC should not be regarded as the ultimate basis of consciousness but are to be seen as macroscopic manifestations of a mechanism underlying conscious systems whose roots lie at a deeper level. This leads to a paradigm shift from a neural substrate of consciousness to a universal substrate of consciousness that certain physical systems can resonantly interact with via a universal coupling mechanism. Systems that are capable of resonant coupling to the ZPF belong to the group of macroscopic quantum systems featuring critical dynamics, with the brain-specific implementation of the coupling mechanism being based on neurotransmitters, particularly on the most abundant neurotransmitter glutamate.

Representatives of class 2 assume that the brain’s electromagnetic field is the substrate of consciousness, with the various branches of this class differing from one another in conceptual terms. One branch postulates an identity between conscious experiences and certain spatiotemporal electromagnetic patterns resulting from neural activity, whereby these patterns are thought to be determined by specific configurations of local field potentials (Pockett, 2000, 2002, 2012). A second branch relies on the hypothesis that consciousness emerges from organized energy, emphasizing that this organization depends on a critical level of resonance (John, 2001, 2002). A third branch views conscious states as inner experiences of information states represented by the brain’s electromagnetic field, provided that the field configurations the information states are based on are sufficiently complex (McFadden, 2002, 2013, 2020). Furthermore, there is a branch that links consciousness to electromagnetic field patterns generated by neuronal assemblies, hypothesizing that the formation of conscious states requires particular organizational and dynamical conditions encountered in collective phenomena and self-organized criticality (Fingelkurts et al., 2009, 2010, 2013). An overview and evaluation of further electromagnetic field theories of consciousness can be found in (Jones and Hunt, 2023).

A common feature of all conventional, classical electromagnetic theories is that they face the challenge of clearly specifying the dividing line between conscious and unconscious field configurations. However, it is precisely this specification that poses an enormous hurdle for classical field theories of consciousness, since the conceptual frameworks underlying these theories cannot provide conclusive answers as to what kind of electromagnetic patterns meet the prerequisites for conscious states, what kind of field configurations qualify as sufficiently complex to transcend the threshold of consciousness, or why the dynamical conditions associated with resonance and criticality should be particularly suitable for generating phenomenal qualities. As already propounded in the discussion of the class 1 theories, the strength of TRAZE is that it can *clearly specify and explain the conditions required for the formation of conscious states*. This is because the conceptual foundations of TRAZE are not based on classical electrodynamics, but on the fundamental theory of the electromagnetic interaction, which is QED. In other words, the transition from the incomplete classical theory of electromagnetism to the complete, fundamental quantum theory of electromagnetism surmounts the obstacles on the way to a self-consistent electromagnetic field theory of consciousness (Keppler, 2021). The key element of QED, missing in classical electrodynamics, is the ubiquitous ZPF, which represents the ultimate substrate of the electromagnetic force. Under the assumption of the dual-aspect nature of the ZPF it becomes obvious why only macroscopic quantum systems that interact resonantly with the ZPF and undergo a phase transition are capable of forming macro-conscious states. This view is compatible with the general resonance theory (GRT) put forward by Hunt and Schooler (2019), which emphasizes the importance of a shared resonance among constituents of the brain and the achievement of a phase transition for the emergence of a macro-conscious entity. Since TRAZE is based on quantum field theory and thus on a deeper description level than GRT, it is possible to substantiate the ideas behind GRT and specify the resonance mechanism as well as the processes underlying a phase transition in greater detail.

This brings us to quantum approaches to consciousness. For quite some time, theories have been discussed that fall into this category, previously designated as class 3. The common element of the major representatives of this class consists in linking conscious processes with quantum state reductions, with the various theories differing in how state reductions are brought about in the brain (Beck and Eccles, 1992; Stapp, 1993; Hameroff and Penrose, 1996, 2014). However, the precise mechanism underlying the formation of concrete conscious states remains a mystery in all these approaches. Ultimately, theories of this kind build on the prevailing interpretation of quantum theory that associates state reductions with the measurement process and in this way introduces consciousness through the backdoor into the quantum-theoretical notion of reality. More recent approaches addressing the foundations of quantum physics point to the conceptual problems concealed behind this interpretation and underline the crucial role of the ZPF in solving these problems (De la Peña et al., 2015), indicating that the ZPF is an indispensable component within the ontological bedrock of quantum theory (Cetto and de la Peña, 2022). In line with these findings, TRAZE postulates the ZPF to be the ontological basis of consciousness and demonstrates that this hypothesis leads to a transparent, well-defined, and comprehensible mechanism behind conscious processes. The key characteristic of this mechanism is the resonant amplification of ZPF modes, which from the double-aspect view of the ZPF can easily be recognized as a necessary precondition for the activation of phenomenal qualities.

In summary, the comparison with other theories suggests that TRAZE offers the conceptual resources to achieve a consolidation of the theory landscape and harbors the potential to evolve into a fundamental theory of consciousness. To drive the further development of TRAZE, a research agenda seems appropriate through which the foundation pillars of the theory are successively reinforced. As it stands today, TRAZE relies firstly on a field-theoretical model of cortical microcolumns that describes the formation of coherence domains, and secondly on coherence-induced downstream effects that follow from the model. The nature of these effects implies that they govern the communication between microcolumns and are crucial for long-range synchronization. This has yet to be proven using detailed quantitative model calculations. Therefore, the communication between microcolumns and the formation of neural activity patterns need to be studied more closely in a future expansion stage of the model. These studies should also include the modeling and deeper understanding of oscillatory network activity, with the aim of providing the QED-based theoretical underpinnings for existing models of large-scale brain dynamics (Deco et al., 2008; Breakspear, 2017). With such a powerful modeling tool at hand, predictions can be made about the dynamical properties of neural activity patterns that may be compared with findings deduced from empirical data. Good agreement between model calculations and data would add additional weight to the hypothesis of the ZPF being the orchestrator of brain activity. This view is fully consistent with the cytoelectric coupling hypothesis, proposing that electric fields guide neural activity, which is supported by data-based analyses (Pinotsis et al., 2023; Pinotsis and Miller, 2023). In parallel to these developments in neurodynamics, the proposition needs to be substantiated that there is a connection between the phenomenal quality associated with a microcolumn and the glutamate concentration determining the frequency of the amplified ZPF modes. This would pave the way for systematic research into the phenomenal structure of the ZPF and for gaining insight into the very nature of consciousness.

## Data availability statement

The original contributions presented in the study are included in the article/Supplementary material, further inquiries can be directed to the corresponding author.

## Author contributions

JK: Writing – original draft, Writing – review & editing.
