# Supplementary material for: Laying the foundations for a theory of consciousness: the significance of critical brain dynamics for the formation of conscious states
Source: Front Hum Neurosci. 2024 Apr 26;18:1379191. doi: 10.3389/fnhum.2024.1379191 (PMC11082359; doi:10.3389/fnhum.2024.1379191)
Supplement: Supplementary file 1 [file Presentation_1.PDF]

Supplementary Material

Laying the foundations for a theory of consciousness: the significance of critical brain dynamics for the formation of conscious states

Joachim Keppler

Appendix A: Summary of the model calculations

The essential steps of the QED-based calculations are presented below. All detailed explanations and relevant references can be found in Section 3 of the article.

The evolution equations of the coupled glutamate-ZPF system can be expressed as

$$\dot{\chi}_0(\tau) = -ig\,\mathcal{A}^*(\tau)\,\chi_1(\tau)\,, \quad \text{(A1a)}$$

$$\dot{\chi}_1(\tau) = -ig\,\mathcal{A}(\tau)\,\chi_0(\tau)\,, \quad \text{(A1b)}$$

$$\frac{i}{2}\ddot{\mathcal{A}}(\tau) + \dot{\mathcal{A}}(\tau) + i\mu\,\mathcal{A}(\tau) = -ig\,\chi_0^*(\tau)\,\chi_1(\tau)\,, \quad \text{(A1c)}$$

where $\chi_0(\tau)$ and $\chi_1(\tau)$ denote the probabilities of occupation of the molecular ground state and preferred excited state, respectively, and $\mathcal{A}(\tau)$ represents the effective amplitude of the dominant ZPF modes. The occupation probabilities obey the normalization condition

$$|\chi_0(\tau)|^2 + |\chi_1(\tau)|^2 = 1\,. \quad \text{(A2)}$$

The coupling strength g and the parameter μ occurring in Eqs. (A1) are given by

$$g^2 = \frac{4\pi}{3}\frac{N}{V}\frac{1}{\varepsilon_0\hbar\omega_0}\,|\vec{D}_{01}|^2\,, \quad \text{(A3)}$$

$$\mu = -\frac{8\pi}{3}\frac{N}{V}\frac{1}{\varepsilon_0\hbar}\sum_{\omega_n\neq\omega_0}\frac{\omega_n}{\omega_n^2-\omega_0^2}\,|\vec{D}_{0n}|^2\,, \quad \text{(A4)}$$

where $\hbar = h/2\pi$ is Planck's constant, ε_0 is the vacuum permittivity, N/V stands for the number of molecules per volume and thus for the concentration of the molecules, and $\vec{D}_{0n}$ represents the dipole matrix elements for the transitions between the molecular ground state $|0\rangle$ and the excited states $|n\rangle$. While g depends only on the preferred vibrational excitation carrying the index $n = 1$, μ includes the complete set of vibrational eigenstates except the preferred excited state. Denoting the energy of the ground state by E_0 and the energies of the excited states by E_n, the resonance frequencies can be expressed by $\omega_n = \frac{1}{\hbar}(E_n - E_0)$. The resonance frequency of the preferred excited state is given by $\omega_0 = \frac{1}{\hbar}(E_1 - E_0)$ and the parameter τ in Eqs. (A1) is to be understood as dimensionless time ($\tau = \omega_0 t$), whereby all time derivatives are also meant as derivatives with respect to τ.

To examine the runaway stage, i.e., the early phase of dynamical evolution, one can apply the initial conditions $\chi_0(0) \approx 1$ and $\chi_1(0) \approx 0$, resulting in the conditional equation

$$\frac{i}{2}\dddot{\mathcal{A}}(\tau) + \ddot{\mathcal{A}}(\tau) + i\mu\,\dot{\mathcal{A}}(\tau) + g^2\mathcal{A}(\tau) = 0\,. \quad \text{(A5)}$$

Since we are interested in an exponential growth of the field $\mathcal{A}$, we are explicitly looking for solutions of the form $\mathcal{A}(\tau) = C\,e^{ip\tau}$ with complex values of p, which leads us first to the polynomial

$$p^3 - 2p^2 - 2\mu p + 2g^2 = 0 \quad \text{(A6)}$$

and finally to the *runaway criterion*

$$g^2 > g_c^2 = \frac{8}{27} + \frac{2}{3}\mu + \left(\frac{4}{9} + \frac{2}{3}\mu\right)^{\frac{3}{2}}\,, \quad \text{(A7)}$$

with g_c standing for the *critical coupling strength.*

For studying the stationary state of the resonant glutamate-ZPF interaction, we make a suitable ansatz to solve the system of coupled differential equations, namely

$$\chi_0(\tau) = \cos\gamma\,e^{i\Theta_0(\tau)}\,, \quad \text{(A8a)}$$

$$\chi_1(\tau) = \sin\gamma\,e^{i\Theta_1(\tau)}\,, \quad \text{(A8b)}$$

$$\mathcal{A}(\tau) = \mathcal{A}_0\,e^{i\Phi(\tau)}\,, \quad \text{(A8c)}$$

which can be demonstrated to satisfy the conservation of probability, energy, and momentum, as required for a stationary state. $\mathcal{A}_0$ is real and positive. The conserved (dimensionless) energy H ($\dot{H} = 0$) is given by

$$H = \frac{1}{2}\dot{\mathcal{A}}^*\dot{\mathcal{A}} + \mu\mathcal{A}^*\mathcal{A} + g\,(\mathcal{A}^*\chi_0^*\,\chi_1 + \mathcal{A}\,\chi_1^*\,\chi_0)\,. \quad \text{(A9)}$$

Inserting Eqs. (A8) into Eqs. (A1) and using Eq. (A9), one finds that the stationary solution must fulfill the following conditions for $\dot{\Phi}$, γ, $\mathcal{A}_0$, and H:

$$\dot{\Phi} = 2g\mathcal{A}_0\cot 2\gamma\,, \quad \text{(A10a)}$$

$$\dot{\Phi} = 1 + \frac{\sin^2\gamma}{\mathcal{A}_0^2}\,, \quad \text{(A10b)}$$

$$H = \frac{\sin^4\gamma}{\mathcal{A}_0^2} + \sin^2\gamma - \frac{1}{2}\,g\mathcal{A}_0\sin 2\gamma, \quad \text{(A10c)}$$

$$(\dot{\Phi}-1)^2 = 1 - \frac{g}{\mathcal{A}_0}\,\sin 2\gamma - 4\,g^2\,\sin^2\gamma\,\frac{(\dot{\Phi}-1)^2}{(\dot{\Phi}-1)^2 - H^2}\,. \quad \text{(A10d)}$$

In company with the coupling strength, Eq. (A3), these equations completely determine the dynamics of the system. The remaining variables are then given by

$$\dot{\Theta}_0 = g\mathcal{A}_0 \tan\gamma \,, \tag{A11a}$$

$$\dot{\Theta}_1 = g\mathcal{A}_0 \cot\gamma \,. \tag{A11b}$$

The establishment of a stationary state is equivalent to the formation of a coherence domain with diameter

$$d = \frac{3}{4}\frac{c}{\nu_0} \,, \tag{A12}$$

which is protected by an *energy gap* of magnitude

$$\Delta E_{gap} = N\hbar\omega_0 H \ = Nh\nu_0 H \,, \tag{A13}$$

where $\nu_0 = \frac{\omega_0}{2\pi}$. Within the coherence domain, the frequency of the dominant field modes is shifted from ν_0 to a lower frequency ν_{CD}, which is determined by

$$\nu_{CD} = \nu_0(\dot{\Phi} - 1) \,. \tag{A14}$$

To perform concrete calculations, the dipole transition matrix elements $\vec{D}_{0n}$ are required, which can be derived from the absorption spectrum of the glutamate molecules. The rewritten equations Eqs. (A3) and (A4) read

$$g^2 = \frac{N}{V}\frac{c}{\nu_0^2}\,\varepsilon_{max}(\nu_0)\,\Delta\nu(\nu_0) \,, \tag{A15}$$

$$\mu = -2\,\frac{N}{V}\,c\sum_{\nu_n\neq\nu_0}\frac{\varepsilon_{max}(\nu_n)\,\Delta\nu(\nu_n)}{\nu_n^2-\nu_0^2} \,, \tag{A16}$$

with $\varepsilon_{max}(\nu_i)$ expressing the peak values of the molar extinction coefficient (one value for each spectral line at resonance frequency ν_i), and $\Delta\nu(\nu_i)$ denoting the widths of the spectral lines (full width at half maximum).

The runaway stage is dominated by the peak concentration of glutamate. Using

$$\left(\frac{N}{V}\right)_{ves} = 300\,\text{mmol/L} = 0.3\,\text{M} \tag{A17}$$

for the vesicular glutamate concentration and taking into account the available data on the absorption lines, one obtains a value pair that fulfills the runaway criterion defined by Eq. (A7). The corresponding values, which arise at the resonance frequency of 7.8 THz, are

$$g_{\,ves} \approx 0.35 \,, \tag{A18}$$

$$\mu_{\,ves} \approx -0.42 \,. \tag{A19}$$

The resonance frequency of the preferred excited state is thus

$$\nu_0 = 7.8\,\text{THz} \,. \tag{A20}$$

The stationary state of the system is governed by the tissue concentration of glutamate. Setting

$$\left(\frac{N}{V}\right)_{tissue} = 12\,\text{mmol/L} = 0.012\,\text{M} \tag{A21}$$

results in a coupling strength of

$$g_{\,tissue} \approx 0.07 \,. \tag{A22}$$

The determinants of the stationary dynamics are then given by

$$\mathcal{A}_0 = 3.845 \,, \tag{A23a}$$

$$\gamma = 0.246 \,, \tag{A23b}$$

$$\dot{\Phi} = 1.004 \,, \tag{A23c}$$

$$H = -0.004 \,, \tag{A23d}$$

which are rounded values. Using Eqs. (A12) and (A20), the diameter of a coherence domain amounts to

$$d = \frac{3}{4}\frac{c}{\nu_0} \approx 30\,\mu\text{m} \,, \tag{A24}$$

and utilizing Eqs. (A14), (A20), and (A23c), a value of

$$\nu_{CD} = \nu_0(\dot{\Phi} - 1) \approx 30\,\text{GHz} \tag{A25}$$

is obtained for the shifted frequency of the dominant field modes within the domain. Due to the huge number of molecules involved ($N \approx 10^{11}$) and the negative value of H, see Eq. (A23d), a coherence domain is protected by a significant energy gap according to Eq. (A13).
